# Strength improvements through occlusal splints? The effects of different lower jaw positions on maximal isometric force production and performance in different jumping types

**DOI:** 10.1371/journal.pone.0193540

**Published:** 2018-02-23

**Authors:** Christian Maurer, Sebastian Heller, Jil-Julia Sure, Daniel Fuchs, Christoph Mickel, Eileen M. Wanke, David A. Groneberg, Daniela Ohlendorf

**Affiliations:** 1 Move functional, Salzburg, Austria; 2 Department of Movement and Exercise Science, Institute of Sport Sciences, Goethe-University Frankfurt/Main, Frankfurt am Main, Germany; 3 Institute of Occupational Medicine, Social Medicine and Environmental Medicine, Goethe University Frankfurt/Main, Frankfurt am Main, Germany; Sao Paulo State University, BRAZIL

## Abstract

**Objective:**

The influence of the jaw position on postural control, body posture, walking and running pattern has been reported in the literature. All these movements have in common that a relatively small, but well controlled muscle activation is required. The induced effects on motor output through changed jaw positions have been small. Therefore, it has been questioned if it could still be observed in maximal muscle activation.

**Method:**

Twenty-three healthy, mid age recreational runners (mean age = 34.0 ± 10.3 years) participated in this study. Three different jump tests (squat jump, counter movement jump, and drop jumps from four different heights) and three maximal strength tests (trunk flexion and extension, leg press of the right and left leg) were conducted. Four different dental occlusion conditions and an additional familiarization condition were tested. Subjects performed the tests on different days for which the four occlusion conditions were randomly changed.

**Results:**

No familiarization effect was found. Occlusion conditions with a relaxation position and with a myocentric condylar position showed significantly higher values for several tests compared to the neutral condition and the maximal occlusion position. Significance was found in the squat jump, countermovement jump, the drop jump from 32cm and 40cm, trunk extension, leg press force and rate of force development. The effect due to the splint conditions is an improvement between 3% and 12% (min and max). No influence of the jaw position on symmetry or balance between extension and flexion muscle was found.

**Conclusion:**

An influence of occlusion splints on rate of force development (RFD) and maximal strength tests could be confirmed. A small, but consistent increase in the performance parameters could be measured. The influence of the occlusion condition is most likely small compared to other influences as for example training status, age, gender and circadian rhythm.

## Introduction

In recent years, several publications have reported an improvement of performance through a change in jaw position [1–10]. Studies have even shown that short-term changes of the occlusion condition induces positive effects on postural control [11–16], body posture [17–22], walking and running patterns [9, 23–25], endurance [8] and strength performance [1, 3, 5, 7, 10]. The myocentric position (the mandible has a balanced isotonic muscle contraction while clinching) has produced more stable postural positions when compared to maximal occlusion condition (habitually relation of the upper jaw to the lower jaw while maximum clenching), centric occlusion, where the jaw condyle is in a centric and rest position (no contact between upper and lower jaw) [12]. In terms of changes in body posture, a slightly more anterior bended position for “angle class II patients” and a more posterior bended position for “angle class III patients” have been described [22]. The angle classification is the classification of dental anomalies after occlusion of the first lower molar compared to the upper molar, whereas angle class II is a distal bite and angle class III is a mesial bite.

Further, Maurer et al. [9] have reported increases in symmetry of kinematics during running. Improvements in endurance have been measured using Wingate test [3], sprints [5] and Cooper test [10] wearing custom-made mouth guards. Furthermore, Ebben et al. [7] have measured improvements of 20% of the rate of force development in countermovement jumps while clenching on a mouth guard. Further, several authors have reported a custom-made mouth guard being more beneficial in terms of the performance measures that were enhanced (e.g. jumping, running speed, peak force, ergometer-tests, back strength) compared to an over-the-counter mouth piece in different types of athletes [2–4, 6, 10, 26]. Reproducible changes in maximal handgrip strength and isometric muscle force of shoulder and quadriceps muscles have been demonstrated within and between days for occlusion conditions [27].

These changes are interesting from an exercise sciences point of view, as maximal strength as well as speed-strength are fundamental basics in various sports [28]. Combinations of different types of jumps have regularly been utilized to easily assess speed-strength [29–32]. Commonly, squat jumps (SJ), countermovement jumps (CMJ) and drop jumps (DJs) from different heights have been performed [33]. The SJ is used to estimate the concentric power, the CMJ to estimate the performance in the slow (> 200ms) stretch shortening cycle (SSC) and the DJs to assess the performance in the fast (<200ms) SSC of muscle chain of the hip and leg extensors. The SSC is characterized for both jumping conditions (CMJ and DJs) by an eccentric phase followed by a concentric phase [28–32, 34]. It seems as if the underlying physiological mechanisms are twofold: 1) during the eccentric phase, tension is applied on the muscle fibres as well as the passive-elastic structures of the muscle-tendon complex. This elastic energy may at least partly contribute to the gain in total impulse observed in the following concentric phase [35]. 2) The lengthening of the muscle fibres during the eccentric phase activates muscle spindles, which then excite the homonymous motoneurons via Ia afferents (= stretch reflex). The resultant reflex activity superimposes the efferent drive and increases the activation of the motor units and therefore increases the impulse output of the muscle [34, 36].

Hitherto, it has not been conclusive at which neural level (e.g. spinal and/or supraspinal) changed occlusal conditions act. Therefore, an assessment of different contraction forms seems useful. Furthermore, it has not yet been shown if the effects are different for the extremities and the trunk and if maximal force production may also be altered.

Two very common orthodontic splints are the Centric splint, in which the jaw joint is positioned in a centric condyle position, and the myocentric splint, in which the jaw is in a myocentric occlusion position (DPS, dental power splint). Complaints in the temporo-mandibular system are often caused by pressing and crunching for which the maximal intercuspidation position (Max) is taken. The Max splint simulates this occlusion position. Therefore, these three splints were used in this study. The neutral position was used as a control condition. The centric (Centric) and the myocentric (DPS) condyle positions are both occlusion positions which are individually adjusted in the centric condylar position. For the Centric splint the focus is on manually finding and fixing the centric condylar position. In contrast the DPS splint is selected by using the transcutaneous electrical nerve stimulation (TENS) system to relax the masticatory muscles [9]. In addition, the materials of the two splints are different: the Centric is manufactured of hard material, while the DPS is made of soft material. The Centric is often used in therapy of temporomandibular disorder (TMD) as a measure in advance of orthodontic, implantological or prosthetic treatment, while the DPS is more often used in the sports sector to increase performance. Therefore, the intention of both splints in dental care is different.

Previous studies have shown the influence of the occlusion conditions on muscle chains [4, 7, 9]. An improvement in balance has been reported, the body posture has become a more upright position and the walking pattern has changed to become more symmetric. The muscle activation in all these movements is generally small. However, in maximal strength tests, it is the opposite: Muscles are maximally activated to produce the highest force or power. It is interesting if small changes induced by a change of the lower jaw position can still cause changes in muscle activation towards a more symmetric or more balanced force production.

The aim of the study was twofold. First, we hypothesised that the two individually joint-related and adjusted occlusion conditions, either in Centric or in DPS position, lead to increases in performance, which are measured as an increase in jumping height (SJ, CMJ), an increase in the performance parameter (DJs), an increase in maximal isometric force and / or rate of force development of the leg extensor muscles, as well as a gain in maximal isometric torque of the extensors and flexors of the trunk. Second, the relationship of muscle forces produced by agonistic and antagonistic muscle chains between the trunk and the abdominal muscles as well as the muscle force relationship between leg extensors of both legs become more balanced using splints.

## Materials and methods

### Subjects

Twenty-three healthy, mid age recreational runners (8f/15m) participated in this prospective study. The mean and standard deviation age was 34.0 ± 10.3 years. In average, the subjects had a weight of 72.1 ± 10.6 kg and a height of 177.8 ± 9.7 cm.

Inclusion criteria were assessed with a questionnaire on the recruitment day [37]. Inclusion criteria were:

- weekly mileage above 25 km, average running speed above 2.7 m/s, running duration per session above 50 minutes;
- no acute pain in the musculoskeletal system that prevents or affects the execution of ordinary activities;
- no abnormalities, traumas or surgeries of the musculoskeletal system as well as in the upper or lower jaw within the last two years;
- no current orthodontic or orthopaedic treatment;
- no locking or abnormal stiffness of the jaw;
- no acute infection, drugs or any vestibular or somatosensory disorder.

Only subjects meeting all inclusion criteria were included in this study. The rights of these subjects were protected and they were thoroughly familiarized with the study design before giving written informed consent to participate in this study, which was approved by the local ethics committee of the medical faculty of the Goethe-University (Nr. 85/ 16) in accordance with the 1964 Helsinki Declaration and its later amendments. The individual in this manuscript has given written informed consent (as outlined in PLOS consent form) to publish these case details.

### Performance tests

#### Jumping tests

Jumping heights and ground contact times were determined with a contact mat (Refitronic, Schmitten (GER)). The manufacturer indicates the measurement error with <0.1%. The jumping heights are determined via the flight time method with the appropriate formula (h = 1/2 g * (t/2)^2^ = g * t2/8; h = S, g = acceleration of gravity [9,81m/s^2^] und t = flight time), while the ground contact time (for the DJ) is measured. Frick et al. [33] calculated a corresponding error estimation.

Drop jumps (DJ) are usually performed from different heights. In our case, box heights were 24 cm [DJ24], 32 cm [DJ32], 40 cm [DJ40] and 48 cm [DJ48]. At the initial position, subjects are standing upright with their hands fixed at the hips on a wooden box. With stepping from the box–horizontally, not downwards–subjects initiate the jump. The movement task is: Try to touch the ground as short as possible and jump as high as you can after the initial ground contact. Single trials only counted to be valid if the subject did not (1) step downwards from the wooden box, (2) loose contact between hands and hips and (3) actively use hip and / or knee joints. For the evaluation of the data, the performance parameter (PP) is calculated, which is calculated as follows: PP = jump height (mm) / contact time (ms) x 100 [38]. The higher the PP, the better the level of performance in reactive force production. The test-retest correlations for the performance parameter from different fall heights have been reported to be between r = 0.85–0.88 (p <0.01 and 0.05, respectively) and can be described as very high [38].

The starting position for the SJ is set to be approximately 90° knee angle with the trunk as upright as possible. The hands are fixed at the hips and all kinds of countermovement (lunge) are prohibited for a valid attempt. Therefore, a purely concentric muscle action produces the impulse. Keiner et al. [32] have reported a test-retest reliability of r = 0.87 (p < 0.01) for the same test [29, 34].

To determine the level of performance in slow-reactive types of contraction, the CMJ has been used. Starting from an upright position, subjects flex their knees to approximately 90° (with the trunk staying as upright as possible with the hands fixed at the hips) before they immediately induce the countermovement. Wirth et al. [34] have reported a test-retest reliability of r = 0.94 (p < 0.01) for the same test.

#### Maximum force of the trunk: Flexion and extension

**Fig 1:**
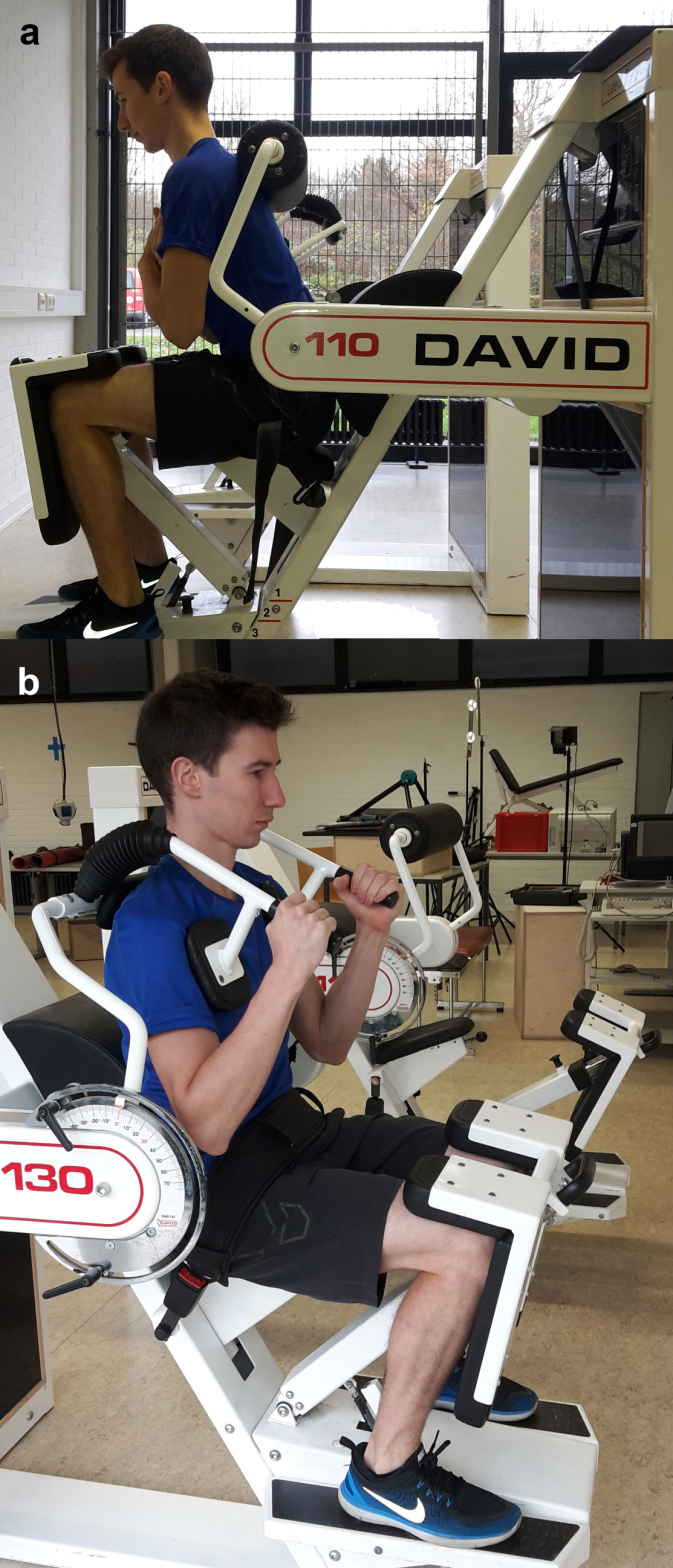
**Illustration of the back extension (a) and back flexion (b)**.

The isometric maximal force testing of the core extensors and flexors was performed using "David 110 and 130" (David Health Solution Ltd., Finland) (Fig 1A and 1B). For the measurements of isometric maximal force, moment arms were mechanically fixed in defined positions. Seat and foot plate height as well as knee fixation were adjusted according to the body size [39] and noted so that all of the data could be recorded in identical positions.

#### Maximum force of the leg muscles

**Fig 2:**
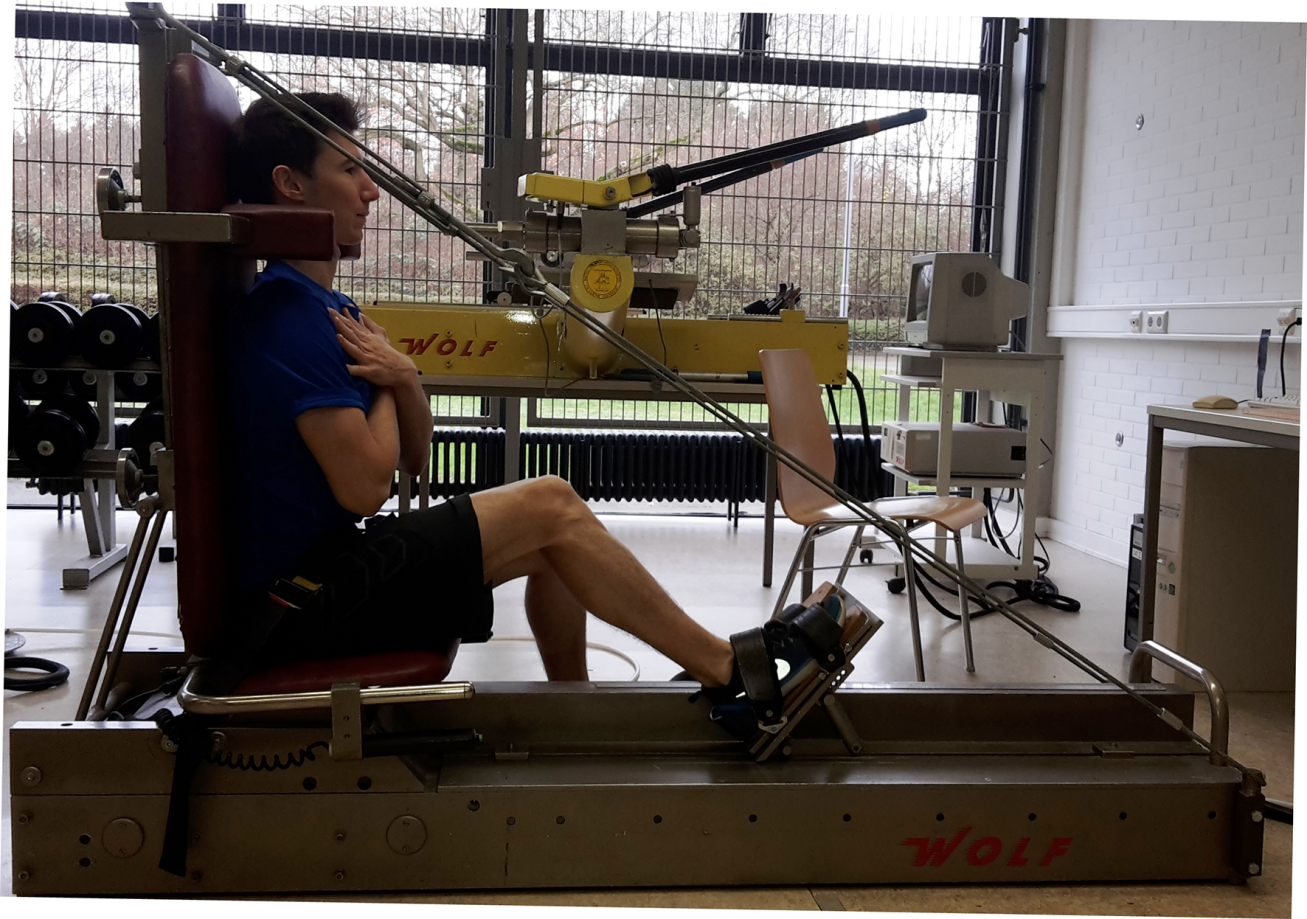
Illustration of the leg press.

The isometric maximal force of the leg extensors was determined using BAG (Wolf, Germany, Test-Retest Reliability r = 0.90; p <0.01) [29]. The manufacturer has reported the overall measuring accuracy being >1% of the end range value, which corresponds to less than 80 N at the maximal measurable power of 8000 N [40]. The measurements were performed in a sitting position and unilaterally with a knee angle of 120°, as this allows for a ballistic movement execution (Fig 2) [40]. Again, the position was noted for the following measurements. Subjects were asked to reach their maximal force production (N) as quickly as possible during each trial and maintain their contraction for approximately three seconds.

### Occlusion conditions

**Fig 3:**
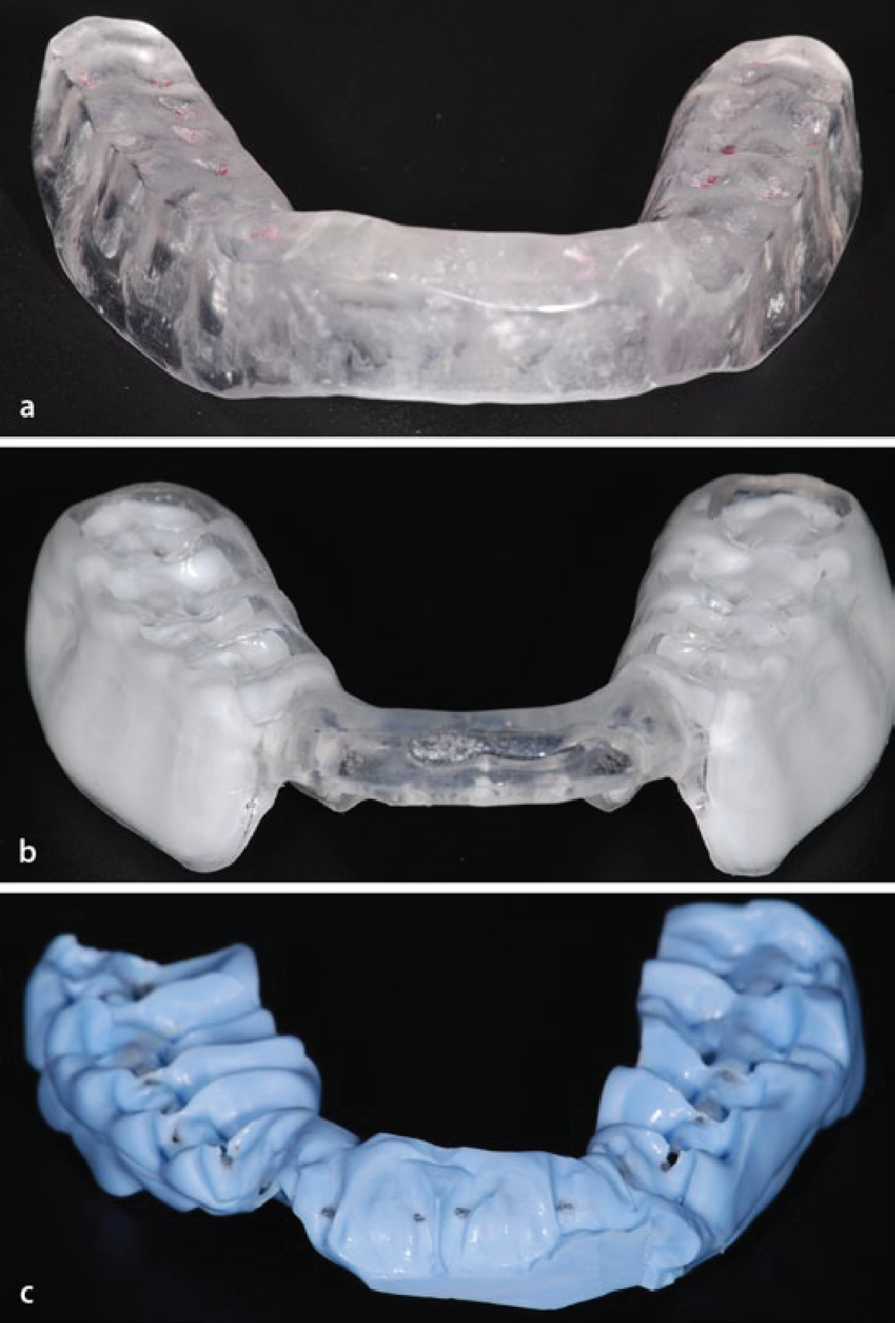
Illustration of the splint conditions. a) Centric, b) DPS, c) Max. A detailed description of the splints is provided in the text.

Based on a previous study [9], the same occlusal conditions were used (Fig 3). Therefore, one condition was the habitual occlusion position at rest (= neutral) [41]; a second was the relaxation splint in centric occlusion (= Centric) [41]; a third was the dental power splint in the myocentric condylar position (= DPS) [42]; and as fourth condition, the maximum intercuspidation splint (= max) was used. Furthermore, a new (fifth) measurement condition was included, which is similar to neutral condition. This "familiarization measurement" (= FAM) was performed before the actual data recording and, therefore, subjects had not yet familiarized with the test protocol.

All three splints (Centric, DPS and Max) were individually manufactured, whereas the Centric and DPS splints are additionally joint-related adjusted. For details on the manufacturing process, please refer to Maurer et al. [9].

### Measurement protocol

The familiarization measurement had always been the first test session, since it was primarily used to familiarize the subjects with the measuring procedures in order to reduce artefacts due to motor learning. Furthermore, subjects performed the test sequence in the four occlusion conditions in random order, again to reduce potential effects of motor learning and training.

Each subject performed their tests on five different days with an interval between measurements of 3 to 7 days to ensure complete regeneration. Additionally, all subjects were instructed to waive moderate training 24 hours and intensive training 48 hours prior to each test session.

Each test session began with a warm-up run for five minutes on a treadmill with the speed set to 2.7 m/s to familiarize with the occlusion condition for that test session. All subjects were instructed to bite on the splint with moderate force during the warm-up and tests. For the test sessions without a splint (FAM and neutral), they were asked to place their tongue on the palate. This instruction was based on the findings of di Vico et al. [43], who have reported significant increases of 30% in knee flexion peak torque at a middle tongue position (thrusting on the lingual surface of incisive teeth) and while the tongue is extended up to the palatine spot.

For the data acquisition, subjects performed each jumping test 5 to 10 times. Maximum force of the trunk was assessed in 3 to 5 trials. Subjects were instructed to slowly and progressively increase torque to reach maximum in 2 to 3 s. For the determination of maximum force production and rate of force development (RFD) of the leg extensors, subjects performed 5 to 10 repetitions. They were instructed to reach peak force as quickly as possible and maintain maximal effort for approximately 3 s. RFD was determined as the steepest increase of the force-time curve. For all maximal force tests, rest between trails was approximately 2 minutes. In-between tests, subjects paused for 5 minutes. For data analysis only the best values of each test and condition was used.

### Statistics

First, the data were tested for normal distribution using the Kolmogorov-Smirnov adaptation test. Group differences between conditions were examined using the Friedman-test. The control for multi comparisons was done using Bonferroni correction for each Friedman-test for each variable. For all test conditions median, 1^st^ and 3^rd^ quartiles are reported. For each subject, the subject’s mean was subtracted to analyse the mean effect of the condition. This was done by calculating the mean over the four conditions for each subject. Then this value was subtracted from the individual results. Median, 1^st^ and 3^rd^ quartile across subjects were calculated and reported. This measure quantifies the median effect of the different occlusion conditions across the subjects.

The Wilcoxon-matched-pairs-test, including Bonferroni correction, was used for paired comparisons between the familiarization measurement and the neutral measurement condition, between both legs and between the back extensors and flexors.

The intraclass-correlation-coefficient (ICC) was calculated from the four measurement conditions to determine whether a larger difference exists between conditions or subjects. The prior Type-I error rate was set at 0.05.

## Results

Data were not normally distributed. No difference was found between the familiarization condition and the neutral occlusion condition for any test condition. The range for the p-value for the different test conditions is: 0.42 to 1. Therefore, the familiarization condition was removed from further analysis.

### Jumping tests

The median [1^st^ quantile/3^rd^ quantile] for the raw jumping values are displayed in Table 1.

**Table 1:** Descriptive data for the different jumping tests and occlusion conditions. For all jumping tests the median [1^st^ quartile/ 3^rd^ quartile] are displayed. The first row in every jumping condition shows the raw values. The second line displays the mean deviation per subject. Significances in the Friedman tests are displayed bold.

		DPS	Max	Neutral	Centric
**SJ [cm]**	**raw**	**28.5 [22.5/34.7]**	**27 [20.4/31.6]**	**28.4 [22/32.2]**	**28.4 [22.8/32.5]**
	**subject mean removed**	**0.7 [0.4/2]**	**-0.5 [0/-2.5]**	**-0.4 [-0.3/-1]**	**0.1 [0/2.3]**
**CMJ [cm]**	**raw**	**29.9 [23.3/35.8]**	**29.1 [22.3/34.9]**	**29.1 [23/34.4]**	**29.7 [23.8/35.3]**
	**subject mean removed**	**0.4 [2/5]**	**0 [-2.5/-4]**	**-0.3 [-1/-3.8]**	**0 [2.3/4]**
DJ24PP [cm/s]	raw	111 [98.5/165]	110 [95.3/150.5]	108 [98.3/155.5]	111 [97.5/158]
	subject mean removed	2 [5/1.5]	-2.5 [-4/-2.1]	-1 [-3.8/-2.8]	2.3 [4/9.5]
**DJ32PP [cm/s]**	**raw**	**131 [108.3/173]**	**116 [101/160]**	**112 [100.5/161]**	**121 [102.5/170.5]**
	**subject mean removed**	**5 [1.5/0.3]**	**-4 [-2.1/1.3]**	**-3.8 [-2.8/-5.5]**	**4 [9.5/1.7]**
**DJ40PP [cm/s]**	**raw**	**124 [109/166]**	**117 [94/164]**	**126 [103.3/164.5]**	**135 [112.5/172.5]**
	**subject mean removed**	**1.5 [0.3/8]**	**-2.1 [1.3/-7.3]**	**-2.8 [-5.5/-7]**	**9.5 [1.7/10.3]**
DJ48PP [cm/s]	raw	140 [112/178.5]	151 [115.3/174.8]	122 [105.8/156]	153 [117/182.8]
	subject mean removed	0.3 [8/0.8]	1.3 [-7.3/-1.3]	-5.5 [-7/-1.5]	1.7 [10.3/3.5]

**Fig 4:**
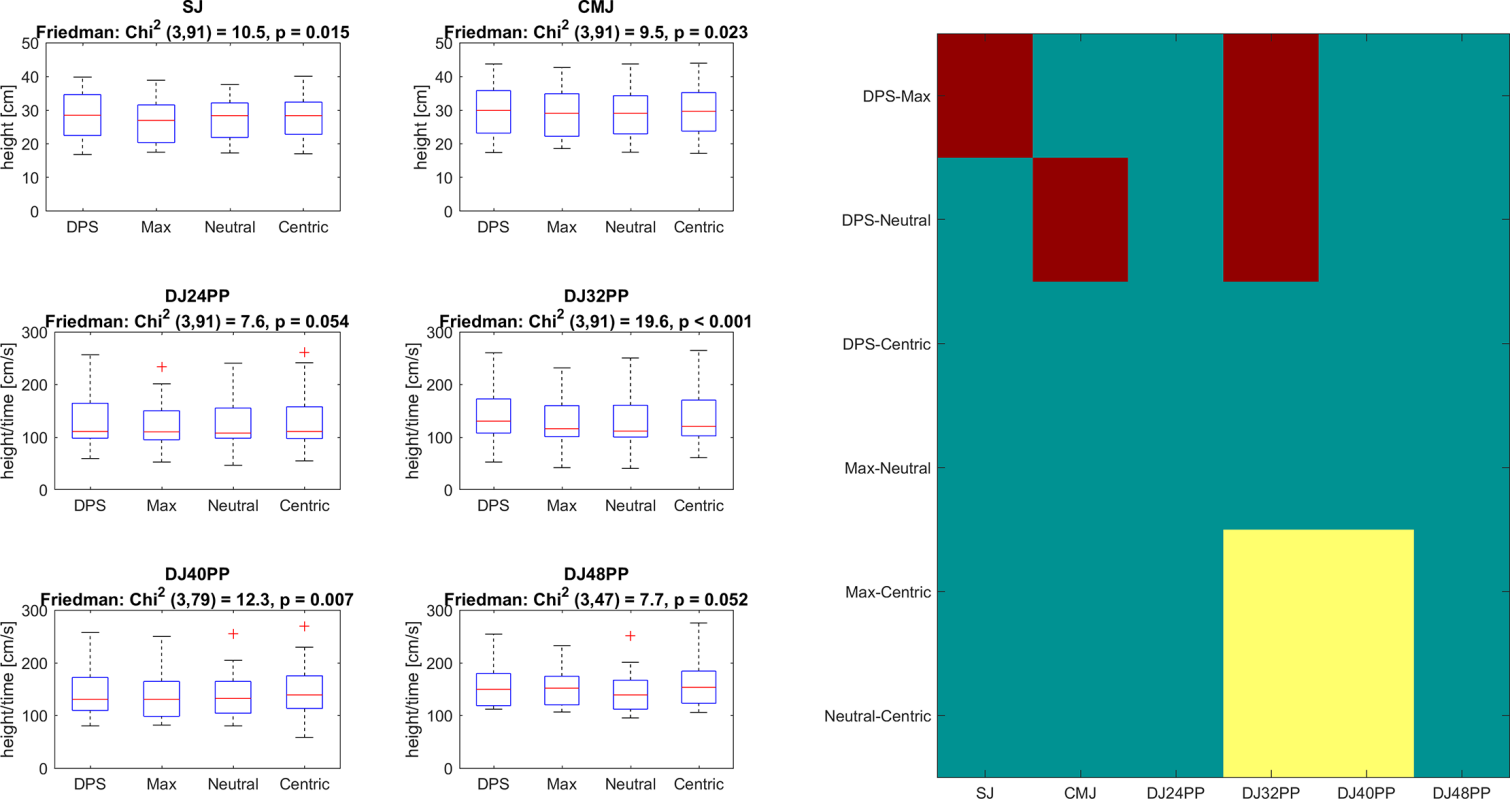
Jumping tests. The 6 graphs on the left side display boxplots for each test. The indiviudal descriptions include both jumping test and Friedman test statistics. The results of post-hoc tests are presented on the right. Significant differences of the mean values (pairwise comparisons) are highlighted in red (dark) if the difference of the two means is positive and in yellow (bright) if the difference is negative. Note that the right hand image was used to indicate the pairpise significant values and was used to increase the readability of the figures.

The Friedman-test revealed significant differences in SJ, CMJ, DJs from 32 cm and 40 cm (Fig 4). Post-hoc analysis disclosed DPS having larger effects than Max for SJ and DJ32 (Fig 4). Furthermore, the effect of DPS was larger than Neutral for CMJ and DJ32, and the effect of Centric was larger compared to Max and Neutral for DJ 32 and 40. No differences were found between DPS and Centric as well as between Neutral and Max conditions.

### Maximum force / torque tests

Median [1^st^ quantile/3^rd^ quantile] for raw maximal force / torque tests are displayed in Table 2. The table is organized in the same way as the table for the jumping conditions. The occlusion conditions are presented in columns. The maximum force / torque tests are presented in rows with the 1^st^ row presenting absolute values and 2^nd^ row showing the values where the subjects’ mean had been removed.

**Table 2:** Descriptive data for the different maximum force / torque tests and occlusion conditions. For all maximum force / torque tests, median [1st quartile/ 3rd quartile] are displayed with the first line presenting raw values and second line displaying the mean deviation per subject. (RFD = rate of force development). Significances in the Friedman are displayed bold.

		DPS	Max	Neutral	Relaxation
**Trunk extension [Nm]**	**raw**	**252 [211.5/298]**	**241 [205.3/271.5]**	**225 [201.5/284.5]**	**236 [208.5/310.5]**
	**subject mean removed**	**8 [0.8/83.9]**	**-7.3 [-1.3/-34.3]**	**-7 [-1.5/-50.8]**	**10.3 [3.5/28.8]**
Trunk flexion [Nm]	raw	180 [136.8/220.8]	173 [141.5/212.3]	180 [136.5/196]	180 [142.8/207.3]
	subject mean removed	0.8 [83.9/0.3]	-1.3 [-34.3/0]	-1.5 [-50.8/-0.1]	3.5 [28.8/0.1]
**Leg press force left [N]**	**raw**	**2274 [1935/2838]**	**2064 [1796/2671]**	**2098 [1706/2714]**	**2248 [1721/2885]**
	**subject mean removed**	**83.9 [0.3/63.1]**	**-34.3 [0/-49.3]**	**-50.8 [-0.1/-37.3]**	**28.8 [0.1/96.3]**
Leg press RFD left [N/ms]	raw	5 [4.2/6]	4.8 [3.8/5.9]	4.7 [3.7/5.9]	5 [4.1/6.1]
	subject mean removed	0.3 [63.1/0.3]	0 [-49.3/-0.3]	-0.1 [-37.3/-0.2]	0.1 [96.3/0.1]
**Leg press force right [N]**	**raw**	**2457 [2149/2982]**	**2260 [1972/2667]**	**2284 [2019/2688]**	**2327 [1943/2993]**
	**subject mean removed**	**63.1 [0.3/0]**	**-49.3 [-0.3/0]**	**-37.3 [-0.2/0]**	**96.3 [0.1/0]**
**Leg press RFD right [N/ms]**	**raw**	**5.2 [4.8/6.2]**	**4.8 [4.1/5.3]**	**4.8 [4/6]**	**4.8 [4.3/6]**
	**subject mean removed**	**0.3 [0/0]**	**-0.3 [0/0]**	**-0.2 [0/0]**	**0.1 [0/0]**

**Fig 5:**
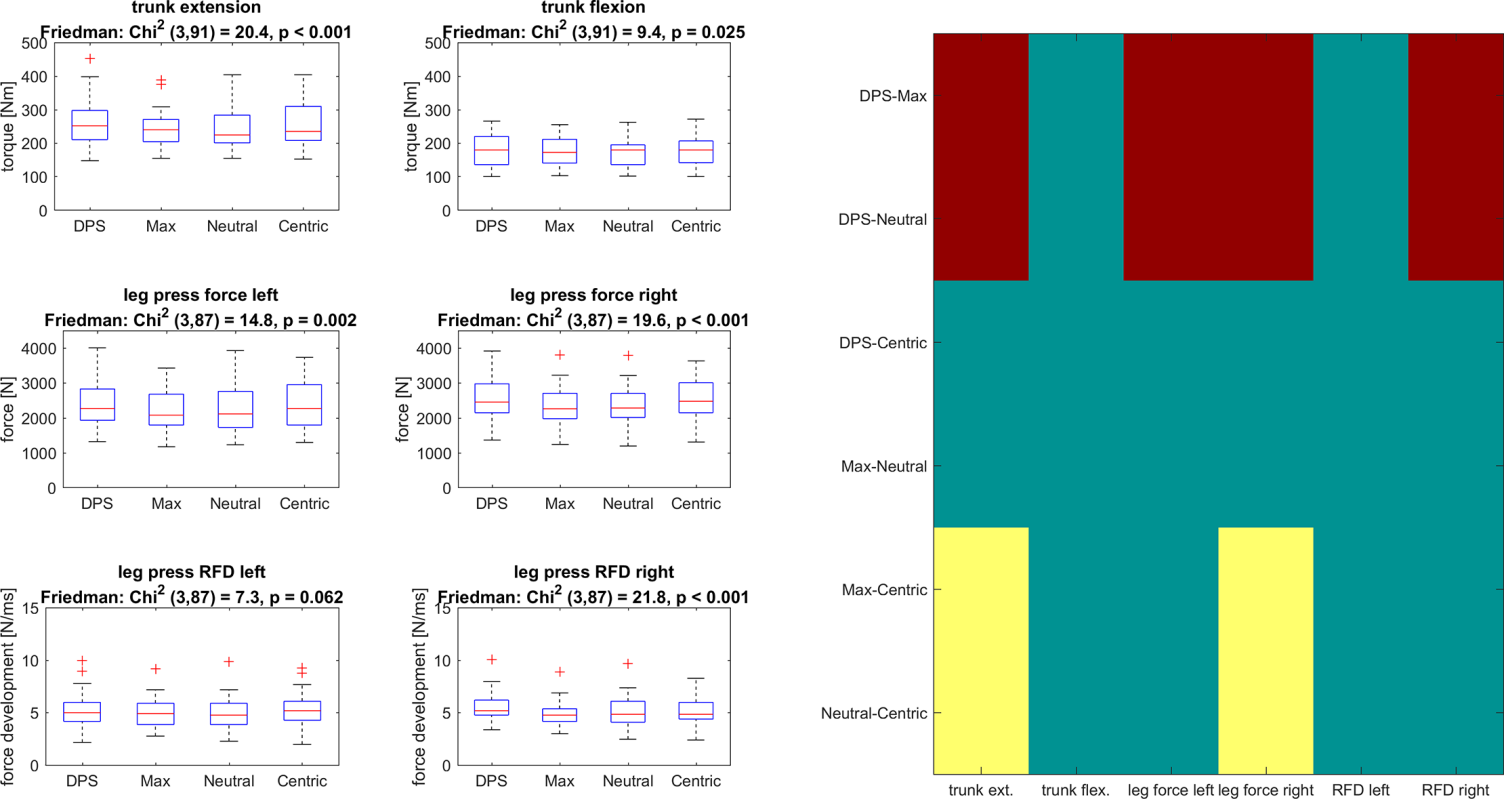
Maximum force / torque tests. The 6 graphs on the left show boxplots for each parameter. The description includes both test and the Friedman test statistics. The results of post-hoc tests are displayed on the right. Significant differences of the mean values (pairwise comparisons) are highlighted in red (dark) if the difference of the two means is positive and in yellow (bright) if the difference is negative. Note that the right hand image was used to indicate the pairpise significant values and was used to increase the readability of the figures.

Friedman-test revealed significant differences in trunk extension, maximal force of leg extensors for both sides and RFD for the right leg (Fig 5). Post-hoc analysis revealed the effects of DPS being larger than Max and Neutral for trunk extension, maximal force of both legs and RFD of the right leg. Furthermore, Centric induced larger effects compared to Max and Neutral for trunk extension and maximal force production of the right leg.

**Fig 6:**
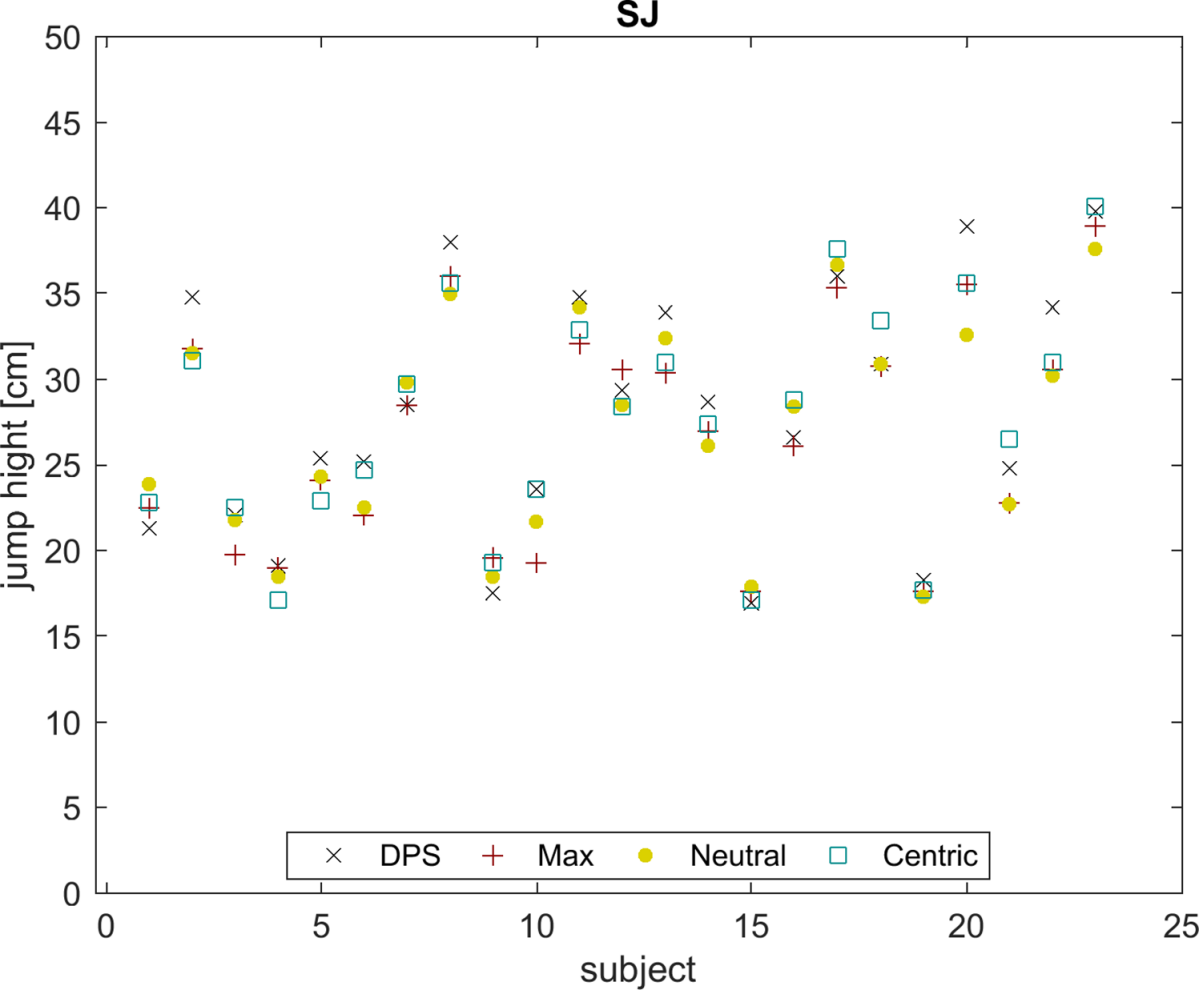
Squat jump–inter- vs. intra-subject variability. Jumping heights of SJ for all subjects and all conditions are plotted. DPS–black cross, Max–red plus, Neutral–yellow dot and Centric–blue square.

Differences between conditions and subjects were assessed via ICC. As shown in Fig 6 for one test, conditions introduce much smaller changes in jumping height compared with the variation between subjects. This holds true for all tests as indicated by an ICC between 0.84 and 0.97. Furthermore, the averaged correlation between the four different occlusion conditions and the 12 tests is 0.07 ± 0.58 (mean ± SD).

### Muscle force relationships

**Fig 7:**
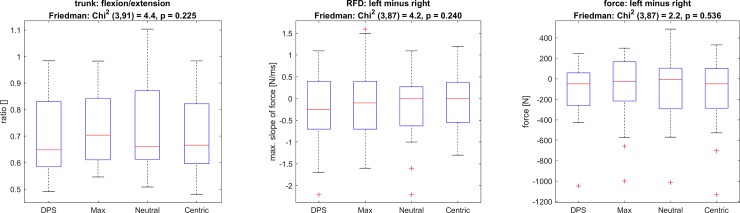
Ratio (flexion/extension) on the right; differences (left minus right) of RFD in the middle; and differences (left minus right) in maximal force on the right.

The relationship between maximal torque production of trunk flexors and extensors as well as comparisons between maximum force and RFD of both legs are visualized in Fig 7. No differences between the four different occlusion conditions were found.

## Discussion

No differences were found between neutral and familiarization condition. Therefore, no motor learning was present and familiarization condition could be removed from further analysis. Further, this result indicates good reliability for all tests.

Consistent differences were found between DPS and Centric as well as Max and Neutral. Those differences concern two of the six jumping tests (speed-strength). For Centric, significances were found for DJs but not for SJ and CMJ. This may indicate a larger influence on fast SSC compared to slow SSC and purely concentric muscle action. However, more data should be collected in order to be able to make a strong conclusion regarding the effectiveness of the Centric splint intervention on specific aspects of neuromuscular function. For the maximal strength tests, DPS showed significantly larger effects than Max and Neutral for four tests, while Centric was significantly superior compared to Max and Neutral for two tests. In total, DPS produced better performance in 50% of the tests compared to Max and Neutral as well as Centric led to better performances in 33% of the tests compared the other two splint conditions.

No differences were found between Max and Neutral as well as between DPS and Centric for speed- and maximal strength. The probability for the twelve tests to result in an always positive relationship for DPS comparisons can be calculated with binomial density distribution. With the four occlusion conditions six possible combinations exist for the post-hoc tests. It was assumed that only three results–negative difference, no significance and positive difference–can be observed. Based on these assumptions, the probability for DPS showing larger effects than two other conditions in 50% of all tests is 0.0001 and for Centric being superior in 33% of the cases is 0.007. Therefore, both results are significant allowing the statement that DPS and Centric lead to a significant increase in the force production. The first research question (H1) can be accepted. However, the level of significance does not give any indication of the magnitude of any beneficial effect.

Allen et al. [1] have reported no changes in CMJ and bench press through an over-the-counter mouth piece. In our study, neither Max nor Neutral induced changes in force production. During the fabrication of the Max splint, no joint related adjustment was made, but only the occlusion in habitual bite position was taken without changing the joint position. This explains the differences to the other two splints. Therefore, our results are in accordance to the results from Allen et al. [1].

Our second hypotheses that individual, joint-related occlusions lead to a more balanced muscle activation must be rejected. No significant differences were found for the ratios of flexion and extension or for the symmetry between left and right legs. According to Denner [39], the relationship for our group should be around 0.65 (male = 0.66; female 0.64). However, the 50% range of this ratio reaches from 0.52 to 0.75. The median of the four occlusion conditions are 0.65, 0.70, 0.66 and 0.67. All of these values are well within the 50% confidence interval, even Neutral. Therefore, it may be hypothesized that our subjects had already a well-balanced ratio beforehand, which could not be further improved. Concerning the symmetry in RFD and maximal force production of the legs, it is interesting to note, that mainly the right side was more affected than the left side. The force production with the DPS splint was higher than the Max and Neutral condition for both legs. The Centric condition seemed to increase only the force production of the right leg. For RFD, the effects induced via DPS was greater than the Max and Neutral condition, however, again only for the right leg. One hypothesis is that the dominant leg—for the majority of subjects this was the right leg–may be more precisely controlled by the nervous system. If the effect of the splint is a change in neuronal activity, this could explain the difference between the left and the right leg. A counter-argument to this hypothesis is the fact that the right and left leg show no difference in force production: t(22) = 1.7 p = 0.10 for Neutral. Previous studies have shown [9] the gait pattern to become more symmetrical using an individually adjusted splint. Our results on symmetry in RFD and maximal isometric force production of the leg extensors as well as the ratio between maximal isometric torque in trunk flexion and extension did not reveal any changes induced through either one of the splints used.

With regard to the inter- and intra-subject comparisons, it becomes clear that in general, differences between conditions are small compared to inter-subject variance for jumping and maximal isometric force / torque tests. According to Fig 6, the changes induced via the different conditions are much smaller than the general distribution of the cohort. The occlusion conditions explain between 2.2% and 13% of the variance between subjects. The effect size of the significant occlusion conditions is in the range between 0.15 and 0.45 (Cohens’d, min and max). Therefore, the influence of the occlusion condition is most likely small compared to other influences as for example training status, age, gender and circadian rhythm. Nevertheless, both individually manufactured occlusion conditions increased speed-strength and maximal isometric force / torque production. Isselee et al. [27] have shown that the influence of the occlusion condition–even if small–is reliable.

The clinical relevance–if any–might be small, meaning that if a force deficit exists, a better occlusion condition in terms of indentation alone might not be sufficient for the deficit to disappear. However, even small changes might be the tipping point if a subject suffers from muscle weakness or temporo-mandibular disorder. Moreover, in elite athletes these small changes might decide on winning or losing, although it needs to be considered that in our study subjects were recreational runners and therefore, performance increases may be different in patients as well as in (elite) athletes. It would be interesting to determine if the effects were different for strength-training experienced subjects.

In our study, subjects had (very) limited time to familiarize to any one of the splints used, as, in general, short-term effects were of interest. It remains an open question how and if a longer familiarization period would lead to different effects in short- as well as long-term. Isselee et al. [24] have reported that a mouth piece leads to reliable results. Therefore, it may be expected that effects would also last for a longer (training) period.

The big question remains: What are the underlying physiological mechanisms that induce the performance increases? First, there were no differences between DPS and Centric in our study. These splints have in common that they adjust the condyle in a more centric position [9], which decompresses the jaw joints on both sides of the body. This should lead to a relaxation of the jaw muscles and consequently to a balanced occlusion in terms of balanced occlusal contact points. Additionally, a compression of the jaw joints can be avoided while biting. Together these factors induce changes in the temporo-mandibular system. Moreover, those neuromuscular changes may be transmitted to the whole body via neural connections as well as active and passive tissues (e.g.: muscle(s) and fascia).

The change of the jaw position alters the sensory signals to the brain. Within this study we tested a jumping condition where a reflectory pathway was involved, and interaction between the reflectory pathway and the modulated state of the premotor cortex due to the changed afferent signals can be speculated. However, changes can also be observed in tests where reflectory pathways are reduced to a minimum as in the SJ and the isometric strength tests. It is well known that transcortical and subcortical pathways exist which can change motor output based on alterations in sensory information [44–47]. This may also be the case for interactions between jaw position and motor output. Nevertheless, it remains unclear, whether the change in sensory condition–introduced by the changed jaw position–results in the observed change of the motor output, or whether a different mechanism is responsible.

In general, the results, which have been reported in the literature, are inconclusive. While some studies have shown positive effects with custom-made splints [3, 6, 10] others did not find any changes [48, 49]. This discrepancy might be due to differences in the manufacturing process of the custom-made splints, as there has not been any international standard. This limitation also holds true for over-the-counter mouthpieces.

## Conclusion

In summary, our results show performance increases in jumping and strength tests for individualized splints, even though they are small (Cohens’d effect size between 0.15 and 0.45) compared to other influences like age, training and sex. Furthermore, no effect on the ratio between maximal isometric torque of trunk flexors and extensors as well as symmetry of RFD and maximal isometric force of both leg extensor chains were found. Therefore, we may conclude that a shift of the condyle to a more central position seems to positively affect strength and speed-strength parameters.
